# Glass eel migration in an urbanized catchment: an integral bottleneck assessment using mark-recapture

**DOI:** 10.1186/s40462-023-00446-6

**Published:** 2024-02-15

**Authors:** A. B. Griffioen, T. Wilkes, O. A. van Keeken, T. van der Hammen, A. D. Buijse, H. V. Winter

**Affiliations:** 1 Wageningen Marine Research, IJmuiden, The Netherlands; 2 Wageningen University & Research, Aquaculture and Fisheries Group, Wageningen, The Netherlands; 3 Deltares, Department of Freshwater Ecology and Water Quality, Delft, The Netherlands

**Keywords:** *Anguilla anguilla*, Recruitment dynamics, Behaviour, Tagging, Regulated water systems

## Abstract

Diadromous fish such as the European eel (*Anguilla anguilla* L.) are hampered by a high density of barriers in estuaries and freshwater systems. Modified and fragmented waterbodies lack tidal flows, and habitat may be less accessible and underutilized compared to free-flowing rivers and estuaries. With rising sea levels and increased occurrence of droughts, the number of barriers may further increase, implying that the need to study migration in such areas may even become more urgent worldwide. To study glass eel migration and behaviour in such highly modified water systems, a mark-recapture study was carried out in the North Sea Canal (NSC) basin, which drains into the North Sea via a large sluice complex. In total, eight uniquely tagged groups (3,797 glass eels) were released near the sluice complex, and 11 groups (2,663 glass eels) were released at inland barriers upstream over a 28 km long stretch in the NSC in spring 2018. The sluice complex attracted 10.3 million glass eel and did not block or delay their immigration. The large and diurnally intensively used coastal ship locks and allowings some saltwater intrusion, efficiently facilitated glass eel migration. Once in the NSC, water outlets from adjacent polders attracted glass eels relative proportional to the discharge of pumping stations. In the NSC, average migration speeds of 0.7 km/day (max. 1.8 km/day) were measured, and this increased with higher temperatures. Redistribution of glass eel from accumulations at inland barriers to other outlet locations was observed in both upstream and downstream directions in the NSC. Passage success and residence time (‘delays’ of 4.1–13.7 days) varied between the different inland barriers. Most of the glass eel, however, appears to settle in the easily accessible habitats within the brackish NSC catchment. This study combined an integral assessment of successive bottlenecks in a modified inland water system.

**Supplementary Information:**

The online version contains supplementary material available at 10.1186/s40462-023-00446-6.

## Background

Diadromous fish species require a sequence of different habitats that are well connected between marine and freshwater habitats to complete their life cycle [1]. However, worldwide, only 37% of rivers longer than 1,000 km remain free-flowing over their entire length, and 23% flow uninterrupted to the ocean [2]. Recent analyses by Belletti et al. [3] showed that 36 European countries have 0.74 barriers per kilometre on average, with the highest densities (> 1 barrier per kilometre) found in central Europe and parts of Western Europe. This has greatly contributed to strong declines in diadromous fish populations in Western Europe [4–6]. Barrier-induced habitat fragmentation and habitat loss threaten diadromous fish populations such as the catadromous European eel (*Anguilla Anguilla* L.) [7–13]. In addition to habitat and connectivity loss, several anthropogenic and natural threats like overexploitation [14, 15] and changes in oceanic conditions and atmospheric regime shifts due to climate change [16–23] are also causes for the strong decline in the current European eel population. Consequently, long-term glass eel density series in the North Sea region show that current recruitment is only 0.4% compared to 1960–1979 [13]. The eel is therefore listed on the IUCN red list as critically endangered [24].

**Fig. 1:**
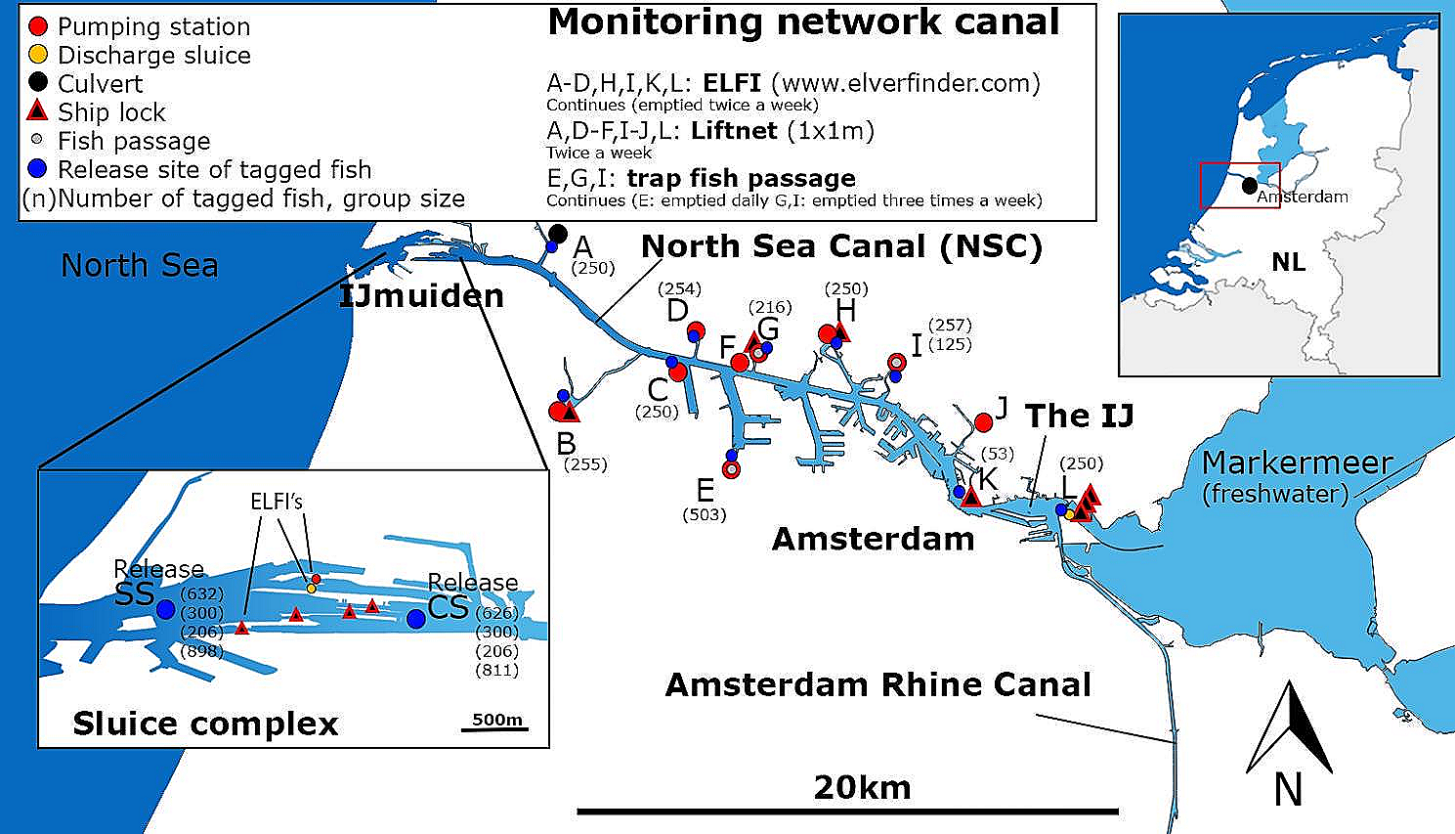
Overview of the study site and locations. SS = IJmuiden Sea Side, CS = IJmuiden Canal Side (CS)

The European eel spawns in the Sargasso Sea and larvae cross the Atlantic Ocean and metamorphose into transparent glass eels when they reach the coastal areas of Europe and North Africa [10]. To reach freshwater ecosystems, glass eels use multiple cues for navigation and guidance (e.g., odours, salinity gradients) including the selective use of tidal flows to colonize freshwater ecosystems [25–29]. In heavily modified systems, however, tidal flows are subdued or cut off by coastal and inland barriers. Barrier-induced habitat fragmentation and a hampered connection between marine and freshwater ecosystems result in less accessible and underutilized habitats for eel. Glass eel migration and habitat utilization in fragmented and heavily modified water systems, including areas below sea level, have been studied far less compared to free-flowing estuaries and river systems [8, 30–35]. With rising sea levels and increased occurrence of droughts due to climate change, the number of barriers and levees in estuaries and hinterlands may even further increase, and the need to mitigate connectivity problems for diadromous fish such as eel may even become more urgent [36]. Highly fragmented and modified areas, such as the Netherlands, are therefore relevant areas to study this general, but urgent, problem of losing tidal dynamics and dealing with subsequent series of barriers from sea to hinterland. The Netherlands, where roughly one-third of the land is below sea level, is managed by an extensive network of dams, dikes, weirs, discharge sluices, pumping stations, ship locks, and drainage canals to prevent flooding. Moreover, the mouths of rivers are blocked by barriers, i.e., dams with sluices, such as lake IJsselmeer (formerly Zuiderzee) and Haringvliet, which are now both large freshwater reservoirs. Additionally, man-made canals were excavated in the Dutch delta for shipping, such as the North Sea Canal (NSC, Fig. 1), connecting Amsterdam to the North Sea during 1865–1876. The NSC has an unnatural stratified salinity gradient along its course. The water level in the NSC is controlled by a complex of ship locks, sluices and pumping stations, forming a large coastal structure that blocks tidal flows and potentially poses a barrier to inland migration.

This study focuses on migration in a highly modified area and studies glass eel migration effectiveness from the North Sea to the brackish NSC and further inland to freshwater polders in the hinterland waters that discharge into the NSC. To our knowledge, this is the first extended mark recapture study following multiple uniquely marked groups in an urbanized catchment. To understand glass eel recruitment dynamics in a highly regulated water system, extensive netting, trapping and mark-recapture experiments were carried out at eleven locations along the NSC to assess the following:

1. Glass eel abundance at the seaside of the IJmuiden complex and at inland potential barrier locations.
2. Passage efficiency and delay at potential barrier sites of glass eels migrating from the North Sea to the NSC and from the NSC to polder areas.
3. Relative distribution of glass eels over inland barrier sites along the canal system in relation to local discharge.

## Materials and methods

### Study area

The study was carried out in the 28 km long North Sea Canal (NSC) from IJmuiden to Amsterdam, The Netherlands, in 2018 (Fig. 1). The NSC drains a basin with a combined surface of over 160 km^2^ [37] containing a network of canals, surrounding polders, and the Amsterdam metropole and connects a large freshwater lake, Lake Markermeer, to the North Sea. A large coastal barrier (sluice complex) at the mouth of the NSC to the North Sea, at IJmuiden, facilitates shipping and water level management and consists of four ship locks (ranging 111–400 m in length and 11–50 m in width), one pumping station (six pumps with total capacity of 260 m^3^/s, being the largest in Europe) and a sluice spilling gate complex with seven gates (max capacity 700 m^3^/s). The ship locks at IJmuiden are operated year-round during day and night to support shipping traffic between the North Sea and Amsterdam and hinterland. The coastal barrier blocks tidal currents from entering inland, and excess water from the NSC catchment is discharged to the sea through the spilling sluice gates around low tide and, when needed, by the pumping station to maintain the NSC at a fixed water level. The average depth in the NSC is 15.1 m and 11.0 m in The IJ (a former estuary at the eastern part of the NSC). Seawater intrudes by lock exchange at the IJmuiden sluice complex into the NSC. This together with the freshwater discharge through NSC results in vertical and a longitudinal salinity gradients with average values of 6.2‰ (range: 4.7–7.4‰) at the surface and 20.6‰ (range: 17.2–26.4‰) near the bottom in the first 18 km from sea between locations CS and I (Fig. 1). In the most inland part near locations J and L, it is 3.6‰ (range: 2.5–4.4‰) at the surface and 14.7‰ (range: 10.9–16.6‰) near the bottom. These average values were derived from a monthly, 2-year long, survey carried out throughout the canal by the national water authority Rijkswaterstaat.

Pumping stations pump excess freshwater from adjacent polders into the NSC. The discharge (m^3^/s) of the pumping stations is registered daily at 5–10 min intervals. All locations (A‒K) are located in connected side canals and harbours near the pumping stations except location C, which is located directly in the NSC (Fig. 1). At location L several ship locks and discharge sluices are present at the transition between the NSC and Lake Markermeer.

### Glass eel sampling and tagging

To monitor and collect glass eel at the study locations, elverfinder traps (‘ELFI’, www.elverfinder.com) and liftnets were used. Also small meshed traps covering the fishways were used at three locations. An ELFI is a mobile glass eel ladder that uses a continuous freshwater attraction flow pumped from the hinterland to attract and trap glass eels. ELFIs were installed on March 20, 2018, and checked twice a week up to July 17, 2018. Traps behind the fish passages were checked three times a week (G and I) or daily (E). In addition, an extensive survey with 1 × 1 m lift nets was carried out at ten locations: A, D, E, F, G, H, I, J, and L, with five hauls at two evenings per week [38]. At two locations (A and E), dipnets were used in addition.

At each study location (Fig. 1), groups of glass eel were caught, tagged and released. Glass eels were anaesthetized with 0.4 ml/l 2-phenoxyethanol and injected in the caudal half of the body with one, two or three small Visible Implant Elastomer Tags (VIE, Northwest Marine Technology). After tagging the glass eels recovered in an aerated tank for a maximum of 24 h. Overall mortality due to tagging or handling was estimated at 0.6%. Combinations of four different fluorescent colours, yellow, red, blue, and orange, were used to create a unique group mark for each batch that was released at a certain time and location (see Appendix A). Glass eels were caught, tagged and released at the IJmuiden sluice complex (SS and CS) as well as at inland barriers along the NSC (locations A, B, C, D, E, G, H, I, K and L).

At the seaside of the IJmuiden sluice complex, 3,979 glass eel were caught in March and April 2018 using liftnets (1 × 1 m and 1.6 × 2.4 m; 1 mm^2^ mesh size) and ELFIs. To assess the total abundance and evaluate the passage success and delay of the IJmuiden Sluice complex, tagged glass eels were released at SS (*n* = 2,036; Fig. 1 and Table B1 in the appendix) and CS (*n* = 1,943) in four paired groups (ranging from 206 to 898 individuals per group) on four different days (March 26 and 29 and April 8 and 16, 2018). The glass eels released at CS were transferred into the NSC directly upstream from the sluice complex.

In addition to the glass eels released at CS and SS, 2,663 glass eels divided into 11 groups (ranging from 53 to 507 individuals per group) were caught, tagged and released at ten locations in the NSC. The glass eels were caught using ELFIs (at locations A, B, C, D, H, I, K and L) and fine meshed (< 2 mm^2^) traps behind fishways (locations E, G, and I). To determine local abundance at inland polder outlets (with pumping stations and locks), to observe dispersal along the NSC and to estimate delay and passage efficiency (at locations E, G, and I), the glass eels were tagged with 11 different colour codes using two or three tags before release at the original catch location (Fig. 1, Table B1). They were released 50–250 m downstream from the subsequent polder outlet sites.

All glass eels caught in each of the samplings were counted and checked for tags by one (ELFIs) or more persons (liftnets and traps behind fish passages). Large catches (> 300 gr) were estimated using total weight and weighing three subsamples of 100 individuals each. Colour reference cards and VI-flashlights (Northwest Marine Technology) were used to determine colour code. All (re)captured glass eels were released at the original catch location, which means that glass eels caught in traps behind passages (locations E, G, and I) were released in the hinterland upstream from the fish passage.

### Overall abundance

To estimate the overall abundance (recruitment) of glass eel approaching the NSC from the sea during spring 2018, the ‘unbiased modified Lincoln-Peterson’ method was used [39, 40] (Eq. 1).

1 $$N= \frac{\left(M+1\right)*(C+1)}{R+1}$$

It was assumed that the ratio between the total abundance (N) and the total number of tagged (M) glass eels is equal to the ratio of the total number of glass eel caught (C) and the total number of recaptures (R). In addition, mixing of the tagged fish among the untagged fish was assumed. The standard deviation was estimated according to [41] (Eq. 2).

2 $${SD= \sqrt{\frac{\left(M+1\right)*\left(C+1\right)*\left(M-R\right)*(C-R)}{{\left(R+2\right)*(R+1)}^{2}}}}$$

If an equal ratio of recaptures between the CS and SS groups was found in the hinterland, then passage efficiency was assumed to be high. If that occurs, then the recaptures of the CS and SS groups can be combined since it was assumed that they have mixed equally among the influx of untagged glass eel. To verify the assumption of equal mixing between tagged and untagged fish, the overall estimated abundance as derived by using combined catches C_all_ and combined recaptures R_SS all_ and R_CS all_ of tagged glass eel released at IJmuiden sluice complex (CS and SS groups) was expected to be similar to estimations using data on recaptures of tagged glass eel released at IJmuiden sluice complex at each of the locations along the NSC separately by using C_local_ and combined recaptures R_SS-local_ and R_CS-local_.

### Local abundance

Local abundances at locations B, C, D, E, F, H, I, K and L along the NSC were estimated using a single batch mark recapture approach. At each location, a single group of locally caught and tagged fish (M) was released to estimate local abundance and average delay. Contrary to the overall abundance approach where several batches divided over the spring period were released, the ratio between C and R (locally released and recaptured glass eels) will change throughout the migration season by glass eels leaving and entering the site. It was assumed that the decrease of the recaptures (R) over time was similar to the decrease of total tagged fish present at the site due to upstream and downstream migrating glass eel at the location (i.e., glass eels leaving the site toward the hinterland or other locations along the NSC). This trend was used to calculate a daily abundance estimate (N_t_) following Eq. 1. To calculate the seasonal (local) abundance, the sum of these daily abundances was corrected for average residence time. The method used to estimate local abundance is further described in Appendix A.

### Passage efficiency at barriers

The efficiency of glass eel passage at the IJmuiden Sluice complex was analysed using recaptures (%) along the NSC of the eight groups and tested by Monte Carlo permutation tests for differences. P values were corrected with Holm’s correction for multiple comparisons. To determine delay at the IJmuiden Sluice complex, migration speed, i.e., the duration and distance between release and recapture time and site, was statistically tested between the CS and SS groups for each release date and combined dates with Monte Carlo permutation tests to test for delay at the IJmuiden Sluice complex. P values were corrected with Holm’s correction for multiple comparisons. Passage efficiencies of three locations (E, G, and I) along the NSC were estimated by the proportion of tagged (M) glass eels released near the passage and recaptures (R) behind the fish passages.

#### (re)Distribution of tagged glass eel

To estimate the number of glass eels that showed redistribution, N_redistributed_. from barriers where they were initially released (i.e., showed movements between different outlet locations), the number of glass eels (R_x_) that showed redistribution from location ‘X’ to location ‘Y’ was corrected for the ‘local catch probability’ to estimate the total number of tagged glass eels that showed redistribution. This was estimated by a local mark recapture experiment using the total number of tagged glass eels (M_y_) released at site ‘Y’ and the number of recaptures of that group (R_y_) according to Eq. 3.

3 $${N}_{redistributed\, x\, to\, y}= \frac{ {\text{R}}_{\text{x}}\text{*}{\text{R}}_{\text{y}}}{{\text{M}}_{\text{y}}}$$

To compare how discharge at each barrier site (attraction flow) was related to local glass eel abundance, discharge (m^3^/s) from pumping stations from the polder into NSC was registered during the study period April 1st – July 17th, 2018.

Analysis was performed using R [42].

## Results

### Delay and passage success from sea to NSC at sluice complex IJmuiden

In total, 709,098 glass eels were caught and checked for VIE markings. Of the 3,979 tagged glass eels released at the IJmuiden Sluice complex either at SS or at CS, 274 glass eels (6.9%) were recaptured at different locations within the NSC (Table 1). Of those, 148 glass eels (avg. 7.3%, between 5.2 and 8.5%) were from the ‘SS-group’ and 126 (avg. 6.5%, between 4.7 and 8.5%) were from the ‘CS-group’. There was no significant difference in the recapture rate of eels released at location CS or SS (*p* = 0.63); therefore, CS and SS recaptures were pooled in further abundance estimates. In addition, at location E, where the highest recapture rate was observed (75%; N_CS_=106 and N_SS_=100), the ratio between the number of recaptures of the CS and SS groups was 1.06, suggesting ~ 100% passage success of the sluice complex.

**Fig. 2:**
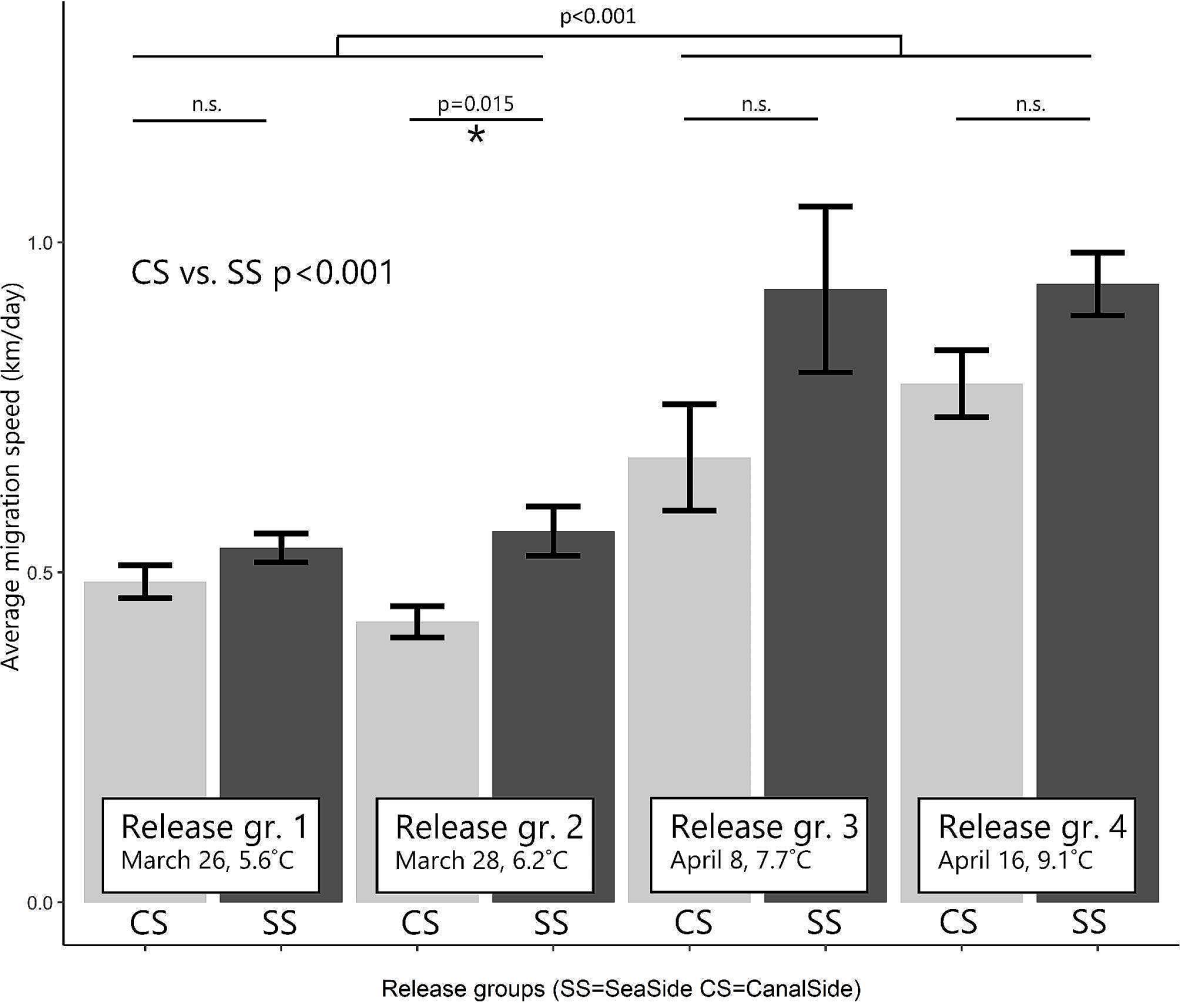
Average migration speed (km/day) of recaptured tagged glass eels released at the IJmuiden Sluice complex either the seaside (SS) or directly behind the barrier at the canal side (CS) on March 26th, 29th and April 8th and 16th, 2018. Error bars indicate standard error; n.s. = no significant difference was found (*p* > 0.05)

Recaptures furthest inland were reported at location L at 26.8 and 29.4 km from the release sites CS (*n* = 1) and SS (*n* = 5), respectively. The average migration speed was 0.7 km/day, and the maximum was 1.8 km/day. The average migration speed was higher in the groups released in April than in those released in March (*p* < 0.001; Fig. 2). The CS groups had a significantly lower migration speed of 0.6 km/day than the SS groups with 0.8 km/day (*p* < 0.001). Of the four different paired CS-SS groups that were released on both sides of the IJmuiden sluice complex, only one showed significantly lower migration speeds for the CS group (*p* = 0.015). This indicates that the delay at the IJmuiden sluice complex was minimal.

**Table 1:** Overall results of the mark-recapture experiment. Total catches (C) per location, recaptures (R) of glass eels of the different groups released at the seaside (SS) and canal side (CS), fish passage efficiency at location E, G and I, average residence time at barriers (delay) and local abundance estimates. * Model assumptions that were used to estimate decrease in tagged glass eels were verified by plotting residuals versus fitted values. In addition, the data were checked for autoregressive temporal correlation. Location I had missing monitoring days after release; therefore, the GLM was not considered to be reliable for that location

		Multiple batch mark recapture experiment throughout the NSC		Single batch mark recapture experiment at multiple locations.					
Location	C (n)	R: SS; CS (n)	R: CS + SS %	Local M	R (ELFI)	R (trap) Passage efficiency	Avg. res. Time (days)	Max. days between release and recapture (days)	Local abundance estimation*
A	3,273	1;0	0.0	250	0 (0%)		n.a.	n.a.	n.a.
B	109,630	12;8	0.5	255	124 (48%)		12.3	65	147,800
C	28,623	9;4	0.3	250	210 (84%)		12.6	59	22,300
D	43,127	15;5	0.5	254	698 (274%)		13.7	75	11,600
E	467,371	100;106	5.2	503	n.a.	79%	4.1	44	580,000
G	9,142	1;0	0.0	216	n.a.	17%	4.4	10	48,000
H	15,974	3;2	0.1	250	210 (84%)		12.0	62	12,900
I	16,714	2;0	0.1	257	84 (33%)	8%	13.4	42	28,800
J	22	0;0	0.0	n.a.	n.a.	n.a.	n.a.	n.a.	n.a.
K	412	0;0	0.0	53	33 (63%)		11.8	56	400
L	14,810	5;1	0.2	250	92 (37%)		11.4	37	29,000
**Total**	**709,098**	**148;126**	**6.9%**				**10.6**		**876,000**

### Glass eel recaptures and overall abundance estimate

The total number of glass eels approaching the NSC at the seaside was estimated at 10.3 ± 0.6 million based on the total glass eel catch (C) and all SS + CS recaptures (R) combined. Separate independent overall abundance estimations using catch and recaptures (originating from CS and SS) of various locations along the NSC varied between 6.5 and 22.2 million glass eels. Sites with estimates that deviated most typically had a low number of recaptures (*n* = 1 or *n* = 2, location G and I) or had potentially missed recaptures due to high numbers of glass eel catches (ELFI at location B.).

### Local abundance and distribution in relation to discharge

**Fig. 3:**
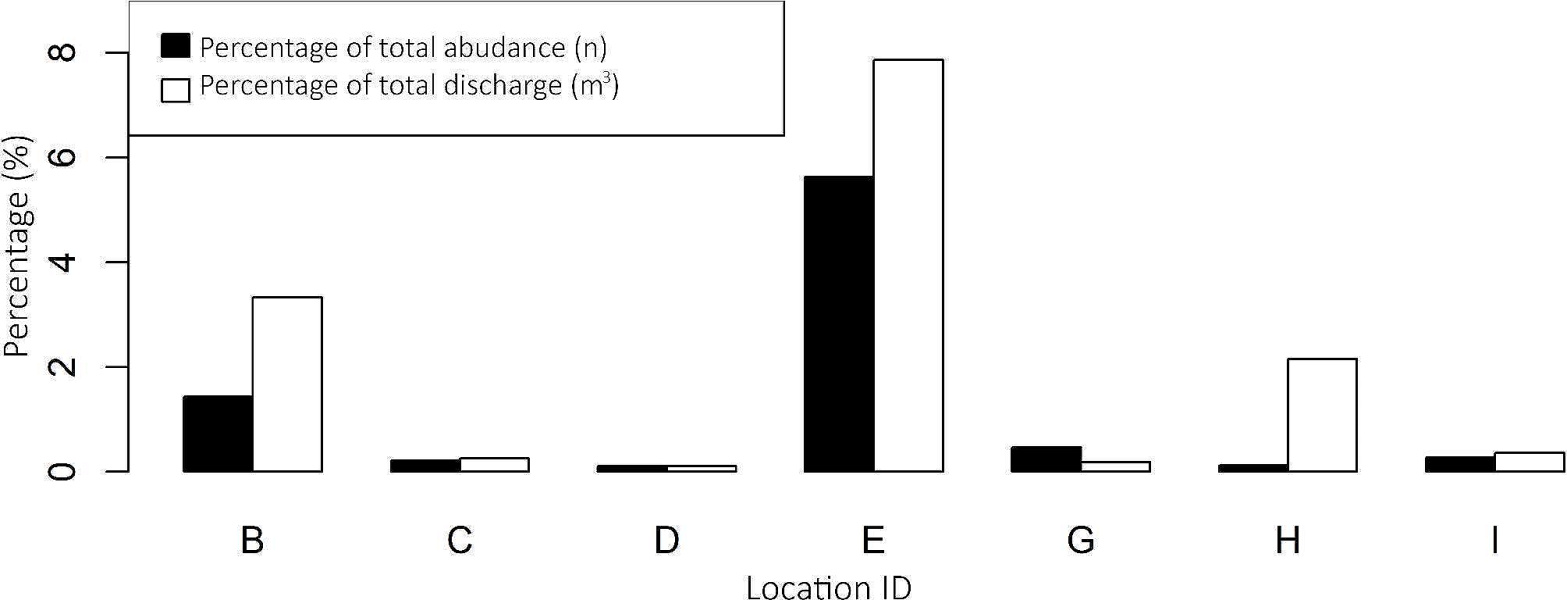
Abundance estimation and discharge as a percentage of total per location

Local abundance estimations ranged between 400 and 580,000 glass eels (Table 1) and combined accounted for 8.5% of the total abundance (10.3 million) entering the NSC. The total discharge of the pumping stations in this study combined was 14.4% of the total discharge of the entire catchment released into the sea. Generally, the higher the discharge is, the more glass eel a pumping station attracts, with a correlation of 0.95. Location H, however, showed much less glass eel abundance than what would be expected based on discharge (Fig. 3). The average discharge during the study period at location H was skewed at and limited to the beginning of April (Figure B1 in the appendix), which may explain the different ratios between abundance and discharge as a proportion of the total. At the other locations, the discharge was more even during the study period.

### Delay and passage efficiency

The average delay was lowest at location E (4.1 days), which also showed the highest passage efficiency of 79% (Table 1). The highest average residence time was estimated at location D at 13.7 days, where glass eels showed prolonged accumulation, resulting in a high recapture rate in the ELFI of 274%, i.e., tagged glass eels were recaptured nearly 3 times on average. In addition, the last locally released tagged glass eel was caught 75 days after release at location D. Other locations with high average delays were locations C (12.6 days, max. 59 days) and H (12.0 days, max. 62 days) and showed prolonged accumulations of glass eels near the inland barrier. The fish passage efficiency was 17% at location G and 8% at location I. Contrary to the other locations, the average delay at location G was short, at 4.4 days, and glass eel were attracted for a short time (maximum 10 days between release and recapture) to this location, probably leaving the site quickly or passing the adjacent ship lock unnoticed.

### Redistribution of tagged glass eel along the NSC

**Fig. 4:**
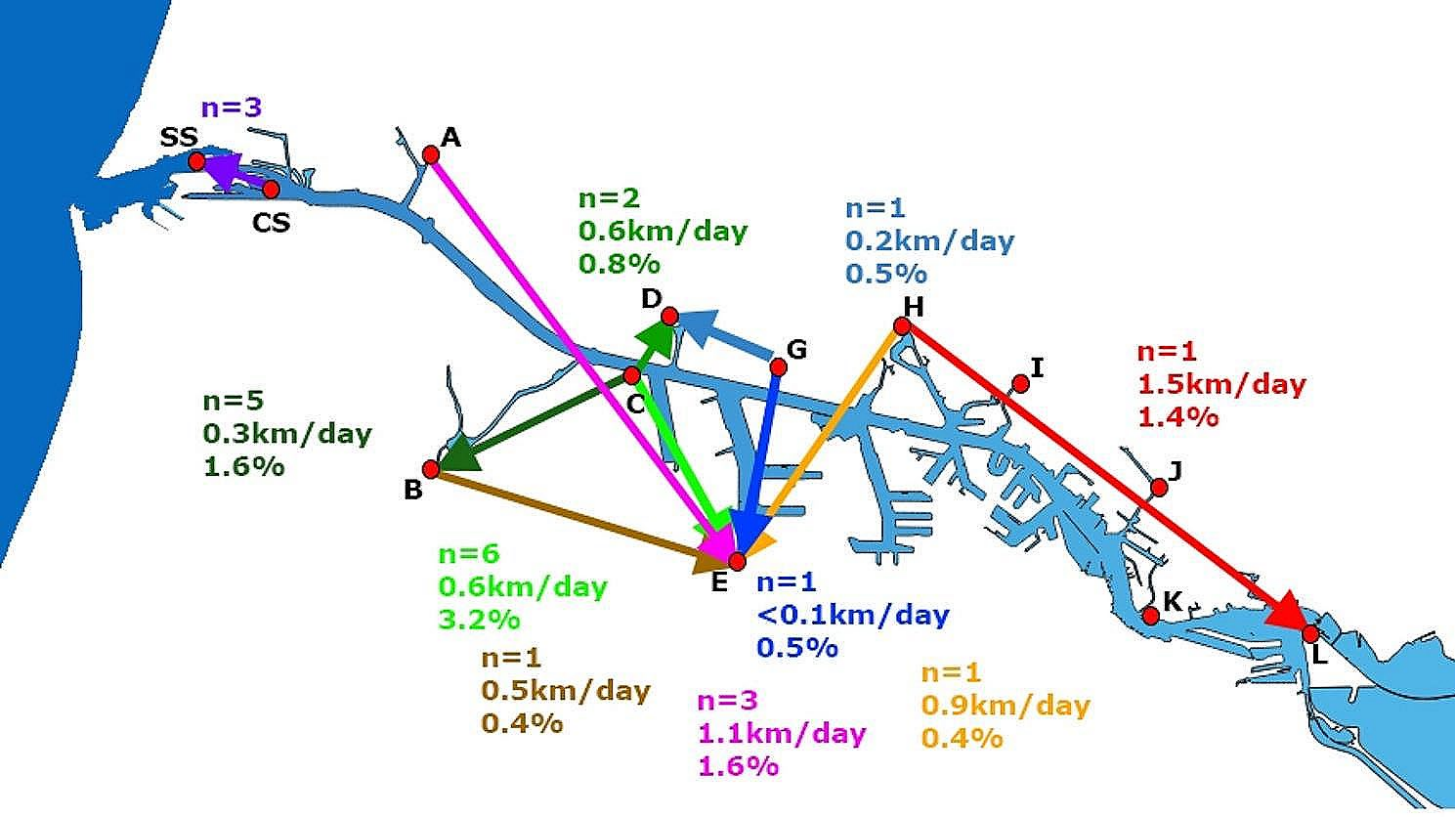
Redistribution of tagged glass eel within the NSC. Each coloured arrow indicates the redistribution from the barrier to the barrier site where tagged glass eel were recaptured corresponding coloured observed numbers of glass eels, average swimming speed and estimated percentage of total dispersal per group corrected for local catch probability

Within NSC, some glass eels showed movements between different outlet locations (‘redistribution’) in all directions: north (*n* = 5), east (*n* = 11), south (*n* = 1) and west (*n* = 4) (Fig. 4). The highest redistribution recaptures were seen originating from location C (contrary to other locations, located directly on the banks of the NSC), 13 glass eels out of 250 tagged glass eels (5.6%). Other locations showed redistribution recaptures between 0.4% and 1.6%. The migration distance between release and recapture varied between 1.8 km (C-D) and 13.6 km (A-E), and the migration speed varied between < 0.1 and 1.5 km day^-1^. When the observed glass eel numbers were corrected for local catch probability and expressed in proportion to local abundance, at least *n* = 2,435 glass eels showed redistribution from locations where they were initially caught and released, i.e., 0.3% of the total abundance at these inland barriers. The highest number of redistributed glass eels corrected for local catch probability (60%) arrived at location E. In addition, three glass eels that were originally released at CS were recaptured at SS, most likely being flushed out by the spilling gates or pumping station or going through the ship locks. At locations I and K, neither glass eel were recaptured from other locations nor were tagged glass eel at these sites observed elsewhere.

### Integral overview of the results

**Fig. 5:**
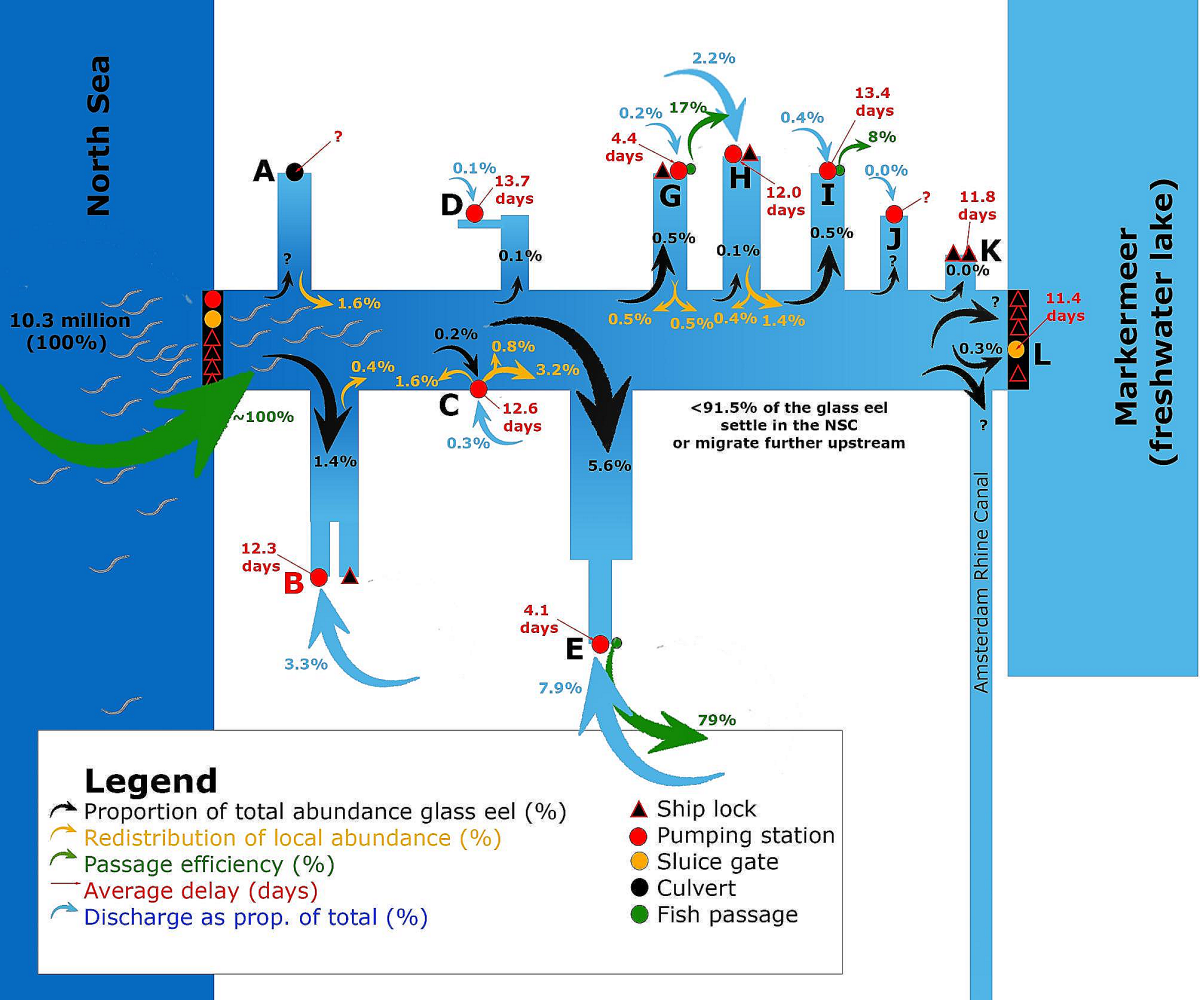
Schematic integral overview of the results of the mark-recapture study in the NSC; relative freshwater discharge (blue arrows), relative glass eel distribution (black arrows) as proportion of the total abundance (10.3 million glass eel entering NSC), passage efficiency (green arrows) and redistribution between NSC outlet locations (orange arrows) are shown

The local abundance of all outlet locations along the NSC combined as proportion to the total abundance explained only 8.5% of the glass eels entering the NSC, suggesting that the majority settled in the NSC itself or in the connected and thus easily accessible habitats of the Amsterdam Rhine Canal, smaller canals of Amsterdam and/or migrated further upstream (Fig. 5). In the NSC, a migratory delay of more than 10 days resulting in accumulations of glass eels was observed at multiple sites (B, C, D, H, I, K and L). In addition to this delay, 0.4–5.6% of the glass eels move to other sites (location A, B, C, G and H). This phenomenon does suggest that glass eel arriving at locations but remain unsuccessful either settle in the NSC itself or search for other areas. Finally, the experimental set up also showed a strong positive correlation between discharge and abundance. This suggests that freshwater discharge (‘attraction flow’) is an important indicator of glass eel distribution.

## Discussion

This mark-recapture study in the NSC, being the entry to a heavily modified catchment of 160 km^2^, using multiple uniquely VIE-tagged groups of glass eel demonstrates that the large sluice complex IJmuiden at the mouth of the NSC did not block or delay the immigration of 10.3 million glass eel that approached this potential coastal barrier. Once inside the canal, which is brackish but lacks tidal currents, water outlets of the polder hinterland attracted glass eels proportional to the discharge through pumping stations. Migration speed significantly increased with higher temperatures (Fig. 2). Passage success, degree of accumulation and delay highly varied between the different outlet sites with pumping stations, sluices and fishways alongside the canal. Redistribution of glass eel was observed in both the upstream and downstream directions. The majority, however, appear to settle in the openly connected habitats within the NSC or move more upstream. The brackish highly regulated environment in the NSC appears to serve as a migration corridor and be suitable for the settlement of glass eel in a similar way as natural estuaries [43–45].

Glass eels passed the IJmuiden sluice complex efficiently without significant delay. Although we cannot determine which route the glass eels used, it is believed that the nightly operation of the ship locks is crucial and efficient to facilitate upstream migration of the glass eels. Migration successes for glass eel highly depend on nocturnal opportunities [46, 47]. As seen in Belgium, nocturnal glass eel migration near tidal barriers can already be successful using short lasting windows when the water levels directly upstream and downstream of the barrier are equal [48]. Although not intentionally managed for this purpose, migration through the ship locks at IJmuiden may function in a similar way as adjusted tidal barrier management, as presented in studies carried out in Belgium [48–50]. A clearly noticeable salinity gradient accompanied by a clearly noticeable but discontinuous local attraction flow and large volumes of water going back and forth through the locks apparently efficiently facilitates glass eel migration.

Considering climate change with accompanying sea level rise [51], an increasing number of summer droughts [52] and salt water intrusion, opportunities for coastal barrier removal or mitigation measures may become scarcer worldwide. To tackle the increasing problem of coastal barriers, insight into factors that enhance the successful immigration of glass eel alongside sluices and shiplocks is needed. Our results suggest that the combination of large volumes, salinity gradients and frequently used noctural shiplocks enabled successful passage. Ship locks along the coast in modified waterbodies may therefore be important for migration. Contrary to coastal ship locks, inland ship locks (e.g., location K) were in operation during the day only and were less effective for attraction. Inland ship locks attracted a lower abundance of glass eel compared to all other locations studied along the NSC. The local attraction flow, including a small salinity gradient in addition to infrequent use and lack of nocturnal openings, seems not to effectively attract glass eel. The pumping station next to the ship lock at location H attracts glass eel on a larger scale, explaining the high abundance near the complex at the ship lock at location H.

This study showed that although inland tidal currents are lacking, glass eels successfully entered the NSC and showed further dispersal to hinterland habitats, as seen by catches in the fish passages and ELFI catches. Glass eels use multiple cues (e.g., attraction flows with salinity gradients and olfactory cues) and migration strategies (e.g., passive drifting, selective tidal stream transport, active swimming) to reach freshwater habitats [27, 53–55]. Due to the lack of tidal currents, distribution in modified water systems in the NSC basin relies on active swimming alone. In the NSC, glass eels showed an average migration speed of 0.7 km/day and increasing speeds with increasing temperatures, with peaks of 1.8 km/day. Glass eels at other sites showed migration speeds of 3–4 km/day in the Gironde basin and 3–5 km/day in the Sevre Niortaise River [25], > 2 km/day in the Ems estuary [56] and 1.6–8.4 km/day for *A. rostrata* in the Penobscot River [26]. In the Cabot Strait, migration speeds were as high as 10–15 km/day [57]. The lower migration speeds in the NSC may be explained by selective tidal stream transport in natural water systems used in estuaries and along the coast, which is lacking in the NSC, where only active swimming can be used for inland dispersal.

A strong correlation between proportional local discharge and local glass eel abundance was found. Attraction flows will be accompanied by the odours of freshwater into the brackish NSC, functioning as additional cues for glass eel [28, 29]. The correlation between discharge and abundance is partially in accordance with Kroes et al. [53], who found that freshwater flows from pumping stations had a significant but small effect on glass eel catch (local density). Contrary to their approach, however, we used total local abundance corrected for residence time instead of local density. Kroes et al. [53] found no significant relation between glass eel densities and freshwater flows at locations with fish passages. The presence of an effective fish passage can drastically reduce residence time and local density over time, as shown at location E in the present study. We found a strong correlation between discharge and abundance. The strongest attraction was found at location E, where the fish passage efficiency was high. Therefore, a large influx of glass eel occurs, resulting in a low density of glass eel. This contrasts with location D, where a high accumulation of glass eel occurred since this barrier had a small (absent) influx (no fish passage). This leads to a high local density of glass eel.

Inland barriers without efficient fish passage could result in prolonged glass eel accumulations. Although limited information is available, predation and loss of condition, due to multiple unsuccessful attempts, might occur at these sites [58–60]. If glass eels fail to migrate to suitable habitats in the hinterland, glass eel might settle in the NSC. It is, however, unknown whether prolonged accumulations and potential condition losses may affect successful settlement, growth, predation avoidance and mortality rates due to density-dependent factors [61].

Mark recapture in addition to recruitment monitoring allows the quantification of total abundance, as used by Diekmann et al. [47]. The use of a non-destructive mark recapture technique VIE tagging, i.e. no fish need to be killed to check for marks and multiple unique group marks can be used, instead of, e.g., destructive methods with only a single group mark available like alizarin red S, is demonstrated to be of additional value to study glass eel behaviour (e.g. determination of delay, redistribution). In addition to measuring local abundance, redistribution between locations was also observed in the present study, which is, to our knowledge, not yet reported elsewhere. To quantify the local abundance of glass eel at the outlet locations, we used one group of tagged glass eel using Lincoln-Peterson with an estimated daily number of tagged glass eel present at the site to calculate the total seasonal abundance corrected for delay. Other studies, however, suggest using multiple groups for abundance estimates [62, 63], which is wise to correct for the variability of environmental factors (e.g., turbidity, moon phase, water temperature) related to glass eel recruitment dynamics [47, 64–66]. Moreover, the position of the barrier (e.g., location C showed more redistribution compared to other locations), the presence or alternative migratory routes (e.g., fish passages or adjacent shiplocks), the water velocity derived from pumping stations and the frequency of pump activity all may vary throughout the season. Therefore, using multiple groups allows for a better analysis of behavioural responses to migratory cues and local delays throughout the season compared to using only one group.

To optimize glass eel migration in highly modified waterbodies, different strategies could be taken. First, the large and diurnally intensively used ship locks in the NSC at IJmuiden successfully facilitated upstream migration of glass eel. Large numbers of glass eels were attracted by a combined flow (pumping station, discharge sluices and ship locks) and guided through the ship locks by a local attraction flow for further upstream migration. In general, ship locks may give inconsistent and only local attraction flows, which might not be optimal for fish migration in general. At coastal barriers, however, a clear salinity gradient at these ship locks may attract glass eels effectively and allow them to attract and facilitate them further upstream. The role of coastal ship locks adjacent to pumping stations and discharge sluices may be as important in other modified catchment areas, including areas below sea level, as at the present study site. Therefore, more insight into factors that determine passage success through ship locks is needed, especially at coastal barriers. Inland ship locks, however, may be less effective. Either by lacking nocturnal operation or the lack of a salinity gradient in combination with low attraction flows. These locks will only facilitate local abundant glass eel if present. Attraction on a large scale at inland sluices is seen if a pumping station is also present, as seen at location H in the present study.

Second, managing attraction flows may be a tool to guide glass eel along the canal and optimize settlement in hinterland and polder habitats. Further research is needed to identify significant differences between attraction flows in relation to cues of the hinterland (e.g., salinity, odours) in modified waterbodies.

Third, the vast majority appear to settle in the connected and thus easily accessible habitats of the NSC, Amsterdam Rhine Canal and smaller canals of Amsterdam. To further study eel settlement in the NSC, analyses of glass eel pigmentation and biometrics of small eels along the canal and along the salinity gradient should be conducted. Glass eel settlement in the NSC may be followed by further dispersal of yellow eels (elvers) in subsequent years [30, 47] and needs attention in fish passage design and seasonal operation.

## Conclusion

This study demonstrates an integral approach to quantify glass eel migration in a highly regulated and modified inland water system. This result showed that a large sluice complex at the mouth of the NSC did not act as a coastal barrier for glass eel passage, but subsequent inland barriers did hamper further upstream passage. With climate change and increasing water levels, coastal water systems will be even more regulated and will affect glass eel migration. The discharge through the sluice complex at IJmuiden attracted large numbers of glass eel (10.3 million). The large and diurnally intensively used ship locks, created noticeable salinity gradients and facilitated unhampered immigration of glass eel with ~ 100% efficiency and no detectable delays. Gaining more insight into the factors that determined this successful passage may aid in finding solutions at other coastal barriers. Subsequent inland barriers, however, severely hampered further migration, which resulted in large areas of potential habitat being underutilized and inducing prolonged accumulations of glass eel with unknown consequences. In modified areas where tidal currents are lacking, glass eels use active swimming and show redistribution in all directions to settle in the hinterland or to migrate further inland. Glass eels were attracted by freshwater flows derived from pumping stations.

### Electronic supplementary material

Below is the link to the electronic supplementary material.

Supplementary Material 1

Supplementary Material 2

## Data Availability

The datasets used and/or analysed during the current study are available from the corresponding author on reasonable request.

## Abbreviations

NSC: North Sea Canal
ELFI: Elverfinder
M: Marked fish
R: Recaptured fish
C: Catch
N: Number of fish
SS: Sea side
CS: Canal side
