# Supplementary material for: Glass eel migration in an urbanized catchment: an integral bottleneck assessment using mark-recapture
Source: Mov Ecol. 2024 Feb 15;12:15. doi: 10.1186/s40462-023-00446-6 (PMC10877867; doi:10.1186/s40462-023-00446-6)
Supplement: Supplementary file 1 — Supplementary Material 1 [file 40462_2023_446_MOESM1_ESM.docx]

**Appendix A**

The trend in the decrease of recaptures R_t_ (expressed in catch per unit of effort, CPUE) was estimated by a generalized linear model (Tweedie distribution) to predict the daily number of tagged fish (M_t_) present following eq. A1.

$$M_{t}=\frac{R_{t}}{R_{t+1}}*M_{t-1}\left( eq.A1 \right)$$

Consecutively, daily abundance (N_t_) was estimated using the ‘unbiased modified Lincoln-Peterson’ method as described in eq. A2. using daily catches C_t_ and daily recaptures R_t_ and estimated number of tagged fish (M_t_). All expressed in CPUE.

$$N_{t}=\frac{\left( M_{t}+1 \right)*\left( C_{t}+1 \right)}{R_{t}+1}\left( eq.A2 \right)$$

To calculate the average delay T_delay_ (residence time at a barrier) the decrease of tagged fish (M_t+1_-M_t_) in a time interval was first multiplied by the number of days between release (t_0_) and time (t) to obtain the total number of days per leaving glass eel. Next, the sum of the total days was divided by the total number of originally tagged fish (M_t0_) to estimate the average delay (eq. A3). The average delay is analysed by an approximation of the total number of tagged fish present based on the recaptures. Some tagged fish however may settle after unsuccessful migratory attempts, therefore ‘delay’ only accounts for the fish that are willing or able to migrate at a location within the study period. Tagged fish that settle may still be in the area but less likely to actively enter an ELFI. Actual residence time may therefore be strongly underestimated.

$$T_{delay}=\frac{\sum\left( \left( M_{t+1}-M_{t} \right)*t \right)}{M_{t0}}\left( eq.A3 \right)$$

Finally, to estimate the average local abundance during the experiment (N_local_), the sum of the daily local abundances ∑N_t_ was divided by the average delay (eq. A4).

$$N_{local}=\frac{\sum N_{t}}{T_{delay}}\left( eq.A4 \right)$$

For locations E and F, the total abundance was estimated with eq. 1 using catch and recaptures in the trap behind the fish passage. This number was verified by dividing the total catch (C) by the percentage of successfully tagged glass eels passing the barrier P_success_ (eq. A5).

$$N_{local}=\frac{C}{P_{success}}\left( eq.A5 \right)$$
