# Supplementary material for: Glass eel migration in an urbanized catchment: an integral bottleneck assessment using mark-recapture
Source: Mov Ecol. 2024 Feb 15;12:15. doi: 10.1186/s40462-023-00446-6 (PMC10877867; doi:10.1186/s40462-023-00446-6)
Supplement: Supplementary file 2 — Supplementary Material 2 [file 40462_2023_446_MOESM2_ESM.docx]

**Appendix B**

**Table B1.** Table of the locations, characteristics of location (SS=seaside, CS=canal side, PS=pumping station, DS=discharge sluices, FP=fish passage and SL=ship lock), catch strategy (ELFI= elver finder, LN = liftnet, DN = dipnet, and trap = trap behind fish passage), release date, time of release, group size and VIE tag colour code.

| Name | Loc. | Location description | | Distance to sea (release SS) km | (Re)capture device | Date release | Time | N | Code |
| --- | --- | --- | --- | --- | --- | --- | --- | --- | --- |
| IJmuiden – group 1 | SS | PS, DS, SL^1^ | | 0 | ELFI, LN | 26 March 2018 | 20:00 | 632 | red |
| IJmuiden – group 2 | CS |  |  | 2.5 |  | 26 March 2018 | 19:35 | 626 | yellow |
| IJmuiden – group 3 | SS |  |  | 0 | ELFI, LN | 29 March 2018 | 11:00 | 300 | blue-red |
| IJmuiden – group 4 | CS |  |  | 2.5 |  | 29 March 2018 | 10:35 | 300 | blue-yellow |
| IJmuiden – group 5 | SS |  |  | 0 | ELFI, LN | 8 April 2018 | 21:00 | 206 | orange-red |
| IJmuiden – group 6 | CS |  |  | 2.5 |  | 8 April 2018 | 19:45 | 206 | orange-yellow |
| IJmuiden – group 7 | SS |  |  | 0 | ELFI, LN | 16 April 2018 | 22:00 | 898 | orange |
| IJmuiden – group 8 | CS |  |  | 2.5 |  | 16 April 2018 | 20:00 | 811 | blue |
| TOTAL | SS |  | | 0 |  |  |  | 2036 |  |
| TOTAL | CS |  | | 2.5 |  |  |  | 1943 |  |
| TOTAL | SS+CS | | |  |  |  |  | 3979 |  |
| Aagtendijk | A | tube | | 7.2 | ELFI, LN, DN | 25 April 2018 | 14:15 | 250 | yellow-yellow-blue |
| Spaarndam | B | PS+SL | | 15.3 | ELFI | 2 May 2018 | 19:15 | 255 | orange-orange-red |
| Houtrakpolder | C | PS | | 12.2 | ELFI | 24 April 2018 | 17:45 | 250 | blue-blue-yellow |
| Nauerna | D | PS | | 14.2 | ELFI, LN | 19 April 2018 | 16:00 | 254 | red-red |
| Halfweg | E | PS+FP^2^ | | 18.0 | trap,LN,DN | 24 April 2018 | 18:00 | 503 | blue-blue |
| Overtoom | G | PS+SL+FP^3^ | | 15.7 | trap,LN | 2 May 2018 | 17:00 | 216 | blue-red-blue |
| Westzanerpolder | F | PS | | 15.5 | LN |  |  |  |  |
| Wilhelmina sluis | H | PS+SL | | 21.7 | ELFI | 2 May 2018 | 17:30 | 250 | orange-orange-yellow |
| de Waker | I | PS+FP^4^ | | 22.0 | ELFI,trap,LN | 24 April 2018 | 18:20 | 257 | yellow-yellow |
| de Waker^5^ | I | " | | “ | " | 5 June 2018 | 17:00 | 125 | orange-orange-blue |
| Kadoelen | J | PS+FP | | 25.4 | LN |  |  |  |  |
| Willem I sluis | K | SL | | 26.0 | ELFI | 22 May 2018 | 16:45 | 53 | orange-blue-orange |
| Oranjesluis | L | SL^1^+FP^6^ | | 29.4 | ELFI, LN | 2 May 2018 | 18:30 | 250 | yellow-red-yellow |
| TOTAL |  | | ALONG CANAL (11 groups) | | |  |  | 2663 |  |

1 In operation 24 hours a day
2 Fish passage with continue attraction flow of appr. 0.2-0.4 m/s during the first six hours of the night.
3 Glass eel ladder with attraction flow between 08:00 PM and 08:00 AM.
4 Fish passage: lock-passage for 5 hours around sunset continues attraction flow (0.4 m3/s). Passage was closed and emptied after 5 hours.
5 Efficiency was studied twice.
6 Fish passage: vertical slot passage

**
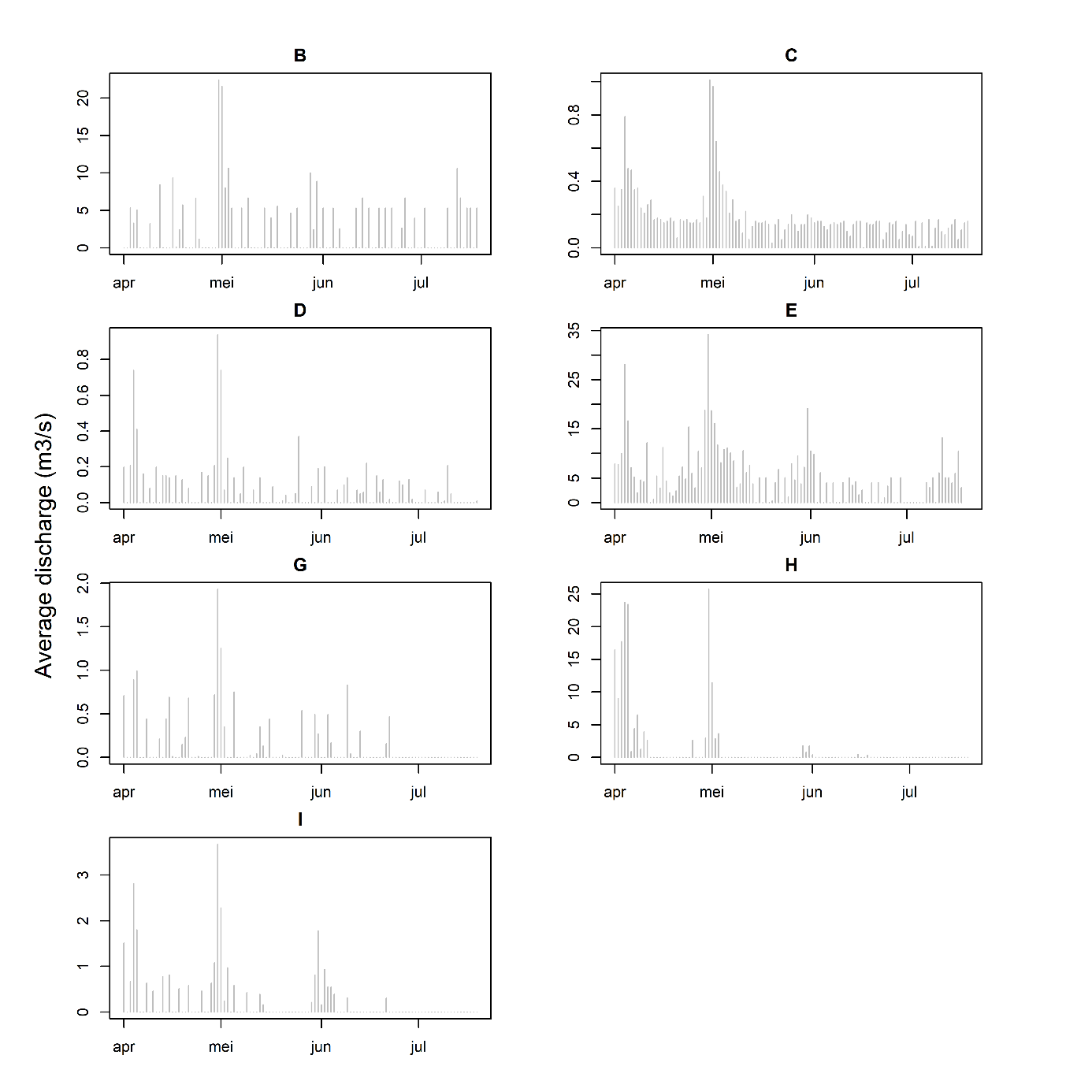
**

**Figure B1.** Average daily discharge (m^3^/s) during the study period April 1^st^ – June 17^th,^ 2018 per location/pumping station.
